# Novel strategy to monitor apparent tumor-specific T cells in patients with cancer

**DOI:** 10.1186/2051-1426-1-S1-P113

**Published:** 2013-11-07

**Authors:** Rieneke van de Ven, Traci L Hilton, Christopher J Dubay, Sachin Puri, Hong-Ming Hu, Rom S Leidner, R Bryan Bell, Walter J Urba, Brendan D Curti, Sandra Aung, Bernard A Fox

**Affiliations:** 1 Earle A. Chiles Research Institute, Portland, OR, USA; 2 UbiVac, Portland, OR, USA; 3 OHSU, Portland, OR, USA

## 

We hypothesize that immune responses that develop following immunotherapy, particularly in patients with objective responses, are directed against a wide range of antigenic determinants. However, in the absence of autologous tumor cells, monitoring is frequently limited to a small number of well-defined tumor-associated antigens (TAA) and underestimates the immune response. Further, we hypothesize that the majority of antigens expressed by HLA on tumor cells are short-lived proteins (SLiPs and DRiPs), which are rapidly degraded by the proteasome, bound to HLA and transported to the tumor’s surface. To develop an antigen source to evaluate this hypothesis in men with prostate cancer, gene expression datasets for prostate cancer (n=85) were compared with several tumor cell lines. One cell line, UbiLT3, had at least 1 overexpressed gene in common (avg 37.1 genes: range 0-200, Z-score 2) with 98.9% of the prostate cancers, suggesting that UbiLT3 had TAA in common with prostate cancer. Next UbiLT3 were cultured with proteosomal and lysosomal inhibitors to accumulate SLiPs and DRiPs in autophagosomes (DRibbles/DRb), which were harvested and used to monitor anti-tumor responses in patients vaccinated with allogeneic prostate GVAX (PC3/LNCaP). After one week of IVS with UbiLT3-DRb, we observed UbiLT3-DRb-specific CD8 T-cell responses in post-vaccine PBMC of 6/12 patients (range 2-22% of CD8). No responses were seen in pre PBMC or healthy donor controls. We could not induce T cells recognizing normal kidney (NK) control DRb after 1-week IVS nor could UbiLT3-DRb stimulated T cells recognize NK-DRb. The difference between pre vs. post DRibble-specific responses was highly significant (p=0.007). To rule out qualitative differences between pre- and post-vaccine antigen-presenting cells, UbiLT3-DRb stimulated CD11c+ cells were isolated from pre and post-vaccine PBMC and were cultured with isolated non-stimulated pre- or post vaccine T cells. Pre and post CD11c+ cells were equally able to induce CD8 responses in post T cells but not pre T cells (n=2). Our data show that vaccination with prostate GVAX induced T cell responses that recognized DRibbles from a cancer cell line that shared common overexpressed genes, but not DRibbles from normal kidney cells. Since no autologous tumors were isolated from these patients, we are looking for ways to evaluate the tumor-killing abilities of these T cells. Current studies are examining this strategy in cancers where autologous tumor cells are available.

## Support

R21 CA123864-02 (WJU), R44 CA121612-02A1 (SA, TH), DAMD 17-03-1-0097 (BAF), Kuni Fnd (BC), OMS (HH, RBB), Robert W. and Elsie Franz, Lynn and Jack Loacker and The Chiles foundation.

